# Tick-borne encephalitis affects sleep–wake behavior and locomotion in infant rats

**DOI:** 10.1186/s13578-022-00859-7

**Published:** 2022-08-02

**Authors:** Gabriele Chiffi, Denis Grandgirard, Sabrina Stöckli, Luca G. Valente, Antoine Adamantidis, Stephen L. Leib

**Affiliations:** 1 Neuroinfection Laboratory, Institute for Infectious Diseases, University of Bern, Friedbühlstrasse 51, 3001 Bern, Switzerland; 2 Graduate School for Cellular and Biomedical Sciences (GCB), University of Bern, Bern, Switzerland; 3 Department of Neurology, Inselspital, Bern University Hospital, University of Bern, Bern, Switzerland

**Keywords:** Tick-borne encephalitis, Langat virus, Infant rats, Sleep, Sleep–wake behavior, Chemokines and cytokines, Neurofilament, Locomotion, Anxiety-like behavior

## Abstract

**Background/Aims:**

Tick-borne encephalitis (TBE) is a disease affecting the central nervous system. Over the last decade, the incidence of TBE has steadily increased in Europe and Asia despite the availably of effective vaccines. Up to 50% of patients after TBE suffer from post-encephalitic syndrome that may develop into long-lasting morbidity. Altered sleep–wake functions have been reported by patients after TBE. The mechanisms causing these disorders in TBE are largely unknown to date. As a first step toward a better understanding of the pathology of TBEV-inducing sleep dysfunctions, we assessed parameters of sleep structure in an established infant rat model of TBE.

**Methods:**

13-day old Wistar rats were infected with 1 × 10^6^ FFU Langat virus (LGTV). On day 4, 9, and 21 post infection, Rotarod (balance and motor coordination) and open field tests (general locomotor activity) were performed and brains from representative animals were collected in each subgroup. On day 28 the animals were implanted with a telemetric EEG/EMG system. Sleep recording was continuously performed for 24 consecutive hours starting at day 38 post infection and visually scored for Wake, NREM, and REM in 4 s epochs.

**Results:**

As a novelty of this study, infected animals showed a significant larger percentage of time spend awake during the dark phase and less NREM and REM compared to the control animals (p < 0.01 for all comparisons). Furthermore, it was seen, that during the dark phase the wake bout length in infected animals was prolonged (p = 0.043) and the fragmentation index decreased (p = 0.0085) in comparison to the control animals. LGTV-infected animals additionally showed a reduced rotarod performance ability at day 4 (p = 0.0011) and day 9 (p = 0.0055) and day 21 (p = 0.0037). A lower locomotor activity was also seen at day 4 (p = 0.0196) and day 9 (p = 0.0473).

**Conclusion:**

Our data show that experimental TBE in infant rats affects sleep–wake behavior, leads to decreased spontaneous locomotor activity, and impaired moto-coordinative function.

**Supplementary Information:**

The online version contains supplementary material available at 10.1186/s13578-022-00859-7.

## Background

Tick-borne encephalitis virus (TBEV) is a positive sense single stranded RNA virus that belongs to the Flaviviridae family causing tick-borne encephalitis (TBE) [1]. Transmission of TBEV is typically through the bite of an infected Ixodes tick [2]. Alternative mode of transmission, through ingestion of unpasteurized milk, can also occur [3, 4]. Three different TBEV subtypes have been identified, namely the European (TBEV-Eur), Siberian (TBEV-Sib), and Far Eastern (TBEV-FE) [2].

Although efficacious vaccines are available, over the last years, TBE has become a growing public health problem in Europe and Asia [5, 6]. The number of human cases of TBE has increased by 400% over the last 30 years [7].

Infection with TBEV-Eur produces a characteristic biphasic disease course. After a short incubation time usually lasting from 2 to 4 days, the first phase occurs (viremia). During the viremic phase of the illness, influenza-like symptoms are observed. Symptoms resolve after 2–6 days. After an asymptomatic interval of approximately 7 days, 45–65% of patients develop neurological signs and symptoms due to infection of the central nervous system (CNS) [8, 9]. The infection of the CNS can manifest in inflammation of the meninges (meningitis), the brain parenchyma (encephalitis), the spinal cord (myelitis), the nerve roots (radiculitis), alone or in combination. In contrast to the TBEV-Eur, the Eastern TBEV subtypes (TBEV-Sib and TBEV-FE) are predominantly monophasic [8, 9]. While the TBEV-FE shows the highest mortality of up to 30%, the TBEV-Eur and TBEV-Sib have much lower mortality rates from 1–2% and 6–8% respectively [2]. There is no specific antiviral treatment available for TBE. Patients often require in-hospital treatment for supportive care depending on the severity of signs/symptoms [4, 10].

TBE may cause long-lasting morbidity, named post-encephalitic syndrome, which significantly affects daily activities and the quality of life. Thirty to 50% of patients after acute TBE develop such post-encephalitic syndrome. Several neurological/neuropsychiatric symptoms have been documented in different prospective and retrospective studies. Most often, cognitive disorders, neuropsychiatric complaints (apathy, irritability, memory, and concentration disorders) as well as sleep–wake disorders are reported, such as altered sleep patterns or fatigue. Furthermore, headache, hearing loss with or without tinnitus, vision disturbances, balance as well as coordination disorders, and flaccid paresis or paralysis are also reported [4, 11–16].

A wide variety of sleep–wake disorders has been reported during the acute phase of TBE as well as during short and long-term follow-ups [17–19]. Unfortunately, only few studies used clear definitions of sleep–wake disorders or assessed them using objective measurement tools or standardized questionnaires. Furthermore, sleep–wake disorders after TBE may well be under-diagnosed or under-reported [14]. However, several studies have shown, that TBE patients suffer significantly more frequently from daytime fatigue compared to age-matched controls [18, 19]. Additionally, newly occurring narcolepsy after the TBE vaccination has been reported [20].

With this study, we aimed to investigate sleep–wake patterns in an established model of TBE using infant rats infected with Langat Virus (LGTV). LGTV and TBEV are two closely related flaviviruses within the mammalian TBEV complex [21]. Within natural environments no diseases caused by LTGV have been recorded in rodents [22]. However, it has been shown that LGTV can be used to induce encephalitis in laboratory mice or rats, when the virus is inoculated intracerebrally or subcutaneously [23, 24]. Infection of mice with TBEV strains usually lead to a very high mortality within few days after infection [24–27], which is not reflecting the clinical situation in humans [27, 28]. Using LGTV, in contrast, induces an attenuated form of the disease that allows to assess long-term effects of an infection. Further, TBEV requires handling under biosafety level 3. The necessity to strictly contain the infected animals precludes the possibility to perform a large panel of behavioral analyses, as planed in the present study, using LGTV.

## Materials and methods

### Animals

All animal studies were approved by the Animal Care and Experimentation Committee of the Canton Bern, Switzerland, and Swiss national regulation for animal protection (License number BE 88/18). A well-established tick-borne encephalitis infant rat model was used [29]. Suckling Wistar strain pups (8-day-old rats with the mothers at the time of supply, Charles River Laboratories, Sulzfeld, Germany) were used in the experiments. Overall, 11 litters were used in the study, containing 113 pups. 58 pups were included in the infection group and 55 in the control group.

### Virus production

The LGTV strain TP21 was kindly provided by Dr. Daniel Růžek (Department of Virology, Veterinary Research Institute, Brno, Czech Republic), and the stocks were prepared by amplification in PC12 rat pheochromocytoma cells for 7 days. The virus stock was then concentrated from culture supernatant by ultrafiltration using an Amicon ultra-15, PLHK Ultracel-PL Membran, 100 kDa (Milipore AG, Zug, Switzerland).

### Virus titration (immunoperoxidase focus assay)

The quantification of the virus concentration was done using an immunoperoxidase focus assay, as previously reported in the literature [30]. The virus was diluted in series and inoculated to 80% confluent Vero cells on a 24 well plate. The plates were placed for 1 h on a rocking platform at 37 °C to allow for viral absorption. The cells were then overlaid with prewarmed cell culture medium (MEM, 1.25% Glutamax, 1% Non-essential amino acids, 1% Antibiotic–antimycotic, 2% FBS, all Thermofischer) containing 1% methylcellulose (Sigma-Aldrich). The plates were incubated at 37 °C and 5% CO2 for 6 days. Cells were washed with PBS pH 7.4, fixed for 1 h with 4% formaldehyde, permeabilized for 5 min with 1% Triton X-100 in PBS, and washed twice with PBST (PBS + 0.05% Tween). The primary antibody (Anti-Flavivirus Group A clone D1-4G2-4–15, Merck-Millipore) was added at a dilution of 1:500 for 1 h at room temperature. The antibody was diluted in blocking buffer consisting of PBST with 10% FBS and 4% Skimmed Milk powder. The cells were then washed twice with PBST and incubated with the secondary antibody (1:500 diluted goat anti-mouse-HRP antibody (anti-mouse IgG (H + L) 1 mg/mL, Sigma-Aldrich, Cat# SAB3701066-1MG) for 1 h. HRP substrate ACE (3-amino-9-ethylcarbozole) (Sigma-Aldrich) in N,N-Dimethylformamide (Sigma-Aldrich) diluted in acetic acid 0.05 M (Sigma-Aldrich) was used to visualize the viral spots after thorough washing. Focus forming units (FFUs) per mL were used to calculate the viral titers.

### Infection of pups

After an acclimatization period of 5 days, rat pups were infected by intracisternal injection of 1 × 10^6^ FFU of the Langat Virus in a volume of 25 µL. As a sham procedure, animals were injected with 25 µL of the PC12 cell medium. The intracisternal injections were performed by using a BD Micro-fine™ 0.3 mL syringe. The injection site was disinfected before injecting the fluid and it was made sure, that the needle was not inserted more than 3 mm. The animals were kept under standard post-op care, consisting of a 2-h close monitoring period after the injection and daily wellness checks afterward.

### Behavioral tests

#### Rotarod test

Animals were subjected to the Rotarod test at day 4, 9, and 21 post infection (pi) in order to assess balance, coordination and physical conditions of the control animals compared to the infected animals. The ability to stay on a rotation drum (Cat. No. 47700, Ugo Basile srl, Comerio, Italy) was tested. The rotating drum was started at a speed of 5 revolutions per minute and accelerated up to 40 rotations per minute over 5 min. The rats were placed individually on the drum and once they are stable, the acceleration started. Latency to fall was then determined. Each animal performed the test three times, and the average time of all three trials was used for the analysis [29].

#### Open field test

The open field test was performed at day 4, 9, and 21 pi in order to assess the spontaneous locomotion. Animals were placed in the middle of a dimly lit arena (45 × 45 × 40 cm) for 2 min. The total walking distance (in centimeters), and the time spent in the center/periphery were determined for each animal using a video tracking system (Noldus EthoVision XT). Each animal repeated the test three times at every time point.

### CSF, blood and brain sampling

CSF sampling was performed on days 4, 9, and 21 pi by puncturing the cisterna magna. Depending on the body weight of the animal between 15 and 30 µL of sample volume was withdrawn. The CSF was centrifuged at 13,000 rpm for 10 min at 4 °C using a table-top centrifuge and the supernatant was transferred into a new tube and stored at − 80 °C until further analysis.

At days 4, 9, and 21 pi, a subset of the animals was sacrificed by intraperitoneal injection of pentobarbital (Esconarkon®, 150 mg/kg, Streuli Pharma AG, Switzerland) for blood and brain sampling.

For the blood sampling, blood was collected from the right ventricle, which was accessed via thoracotomy. Blood was collected using a 23-gauge needle, and 0.5–1.0 mL were collected. Blood samples were placed at room temperature for 30 min enabling clotting. After 30 min the samples were centrifuged in a refrigerated centrifuge (4 °C) for 10 min at 2000×*g* and serum was collected and transferred into a new tube and stored at − 80 °C until further processing.

To harvest the brains, animals were perfused with 25–30 mL ice-cold PBS using a 25-gauge butterfly needle, inserted into the left cardiac ventricle, while an incision was made at the right atrium. The brain was then removed and placed on dry ice and stored at − 80 °C.

### Implantation of the EEG and EMG electrodes

**Fig. 1:**
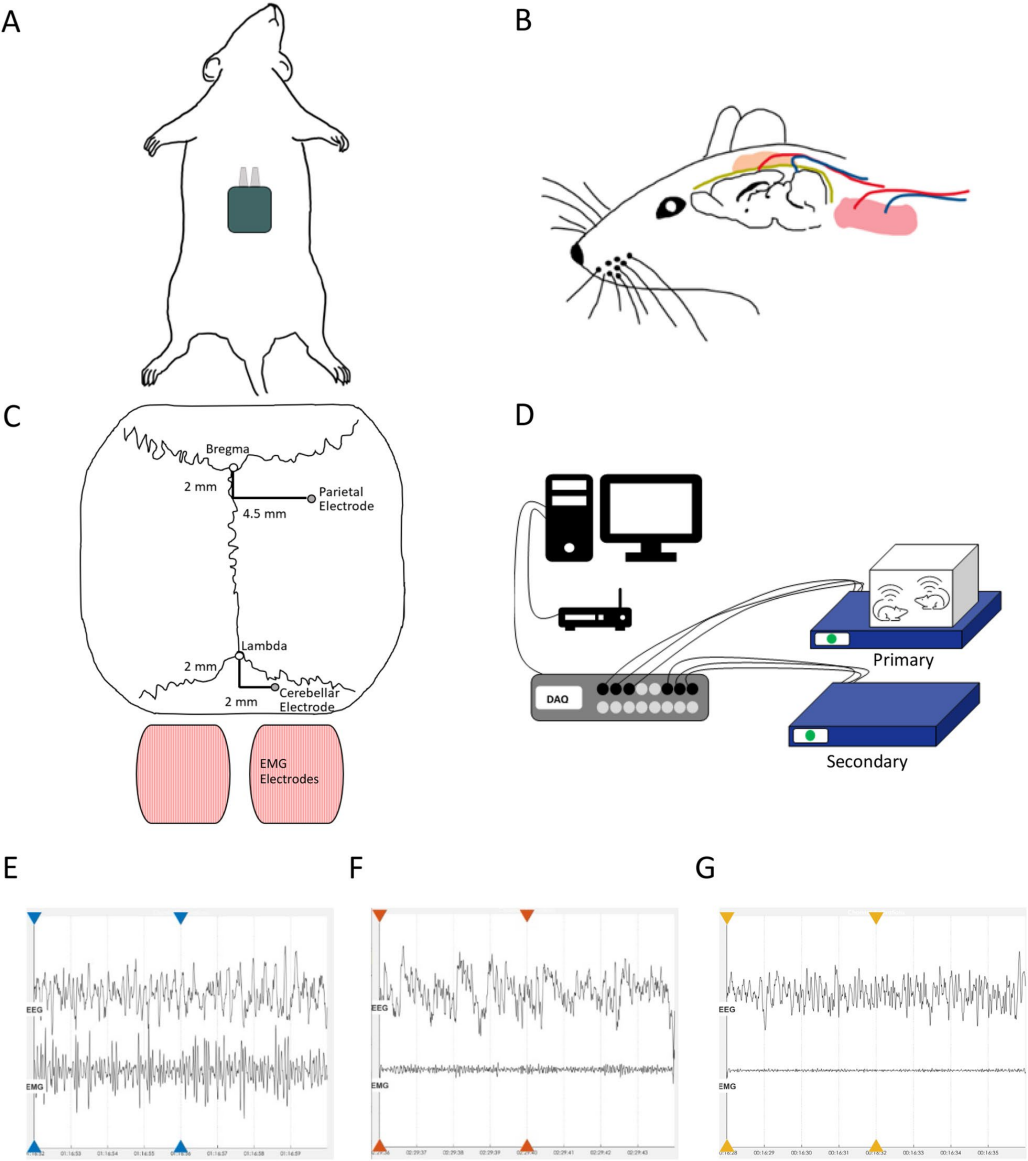
Experimental setting for Telemetry and EEG/EMG recording and exemplary labeled EEG/EMG data: **A** the placement of the telemetry body in the abdomen. **B** Representation of an animal head with implanted EEG and EMG electrodes. **C** Electrode (EEG and EMG) placement in the skull and neck muscle of the animal (not to scale). **D** Schematic displaying EEG/EMG recording. Two stacked SmartPads are used to allow Cohousing in a cage. The primary SmartPad is needed for wireless power to the implanted telemeters whereas each implanted sensor transmits the data separately through the two pads. **E** Exemplary data of a Wake stretch, which is characterized by high and variable EMG activity and medium EEG activity. **F** Exemplary data of a NREM sleep phase, characterized by low EMG activity and prominent EEG activity in the delta range. **G** Example of a REM sleep phase characterized very low EMG activity and medium EEG activity mainly in the theta band

The telemetry EEG and EMG electrodes were implanted following the manufacturer’s instructions. As such animals were anesthetized with isoflurane in oxygen (5% for induction, 1.5–2.5% for maintenance). Under deep anesthesia abdomen, neck, and head of the animal were shaved. On the shaved sites Betadine® was applied to avoid postoperative infection and Lidocaine (Lidocain HCI “Bichsel” 0.5%, 5 mg/kg) was applied locally subcutaneous (s.c.) at the site of the incision before surgery. Under aseptic conditions, the telemeter body (Kaha Sciences, TR50BB) was implanted in the abdominal cavity and secured to the abdominal wall using the suture tabs on the telemeter body (Fig. 1A). Once the telemeter body was secured the abdominal wall was closed with sutures (“Silk”, black silk 1,5 (4/0), 100 m, Dieckhoff & Ratschow Praxisdienst GmbH &Co.KG, Germany). A subcutaneous blunt dissection was performed to the nape of the neck followed by a small incision. The electrode leads were then threaded through the trocar and externalized at the neck. Before implanting the EMG electrodes, blunt dissection to expose the nuchal muscle was performed. The EMG electrodes were secured in place on the ipsilateral nuchal muscle with a suture with their extremities placed into the muscle (Fig. 1B).

A midline incision through the scalp and clean connective tissue from the skull was performed. Holes were drilled using a 0.9-mm steel drill (1RF 009, Meisinger, Germany). One electrode was implanted in the dura mater of the parietal cortex (2 mm posterior of the bregma and 4.5 mm lateral to the midline in the right), and the second in the cerebellar cortex (2 mm posterior of the lambda and 2 mm lateral to the midline in the right) as described in [31] (Fig. 1C). For securing the electrodes dental cement (Vertex™ Self Curing number 3, 1000gr; Vertex Self Curing liquid 1000 mL, ITD Intertrading Dental AG, Switzerland) was applied on the skull. As an anchor for the dental cement two screws (1.1 mm O.D. × 1/8 inch, F00CE125, J.I. Morris) were implanted in the skull.

After implantation, incisions were closed using wound clips (Autoclip Wound Closing System – Clips 9 mm). Postoperative analgesia was provided after surgery and for the three following days (Metacam® 5 mg/kg, daily, s.c., Boehringer Ingelheim Vetmedica GmbH, Germany) and animals were monitored twice a day until EEG measurement.

### EEG measurement and scoring

EEG and EMG from each animal were measured for 24 consecutive hours on day 39 p.i. (Fig. 1D), with two animals co-housed in a cage. The sleep measurements were done scored for Wake, NREM and REM epochs. In order to do so, a two-stage process was used. As a first step, a previously published algorithm was used for automatic scoring [32] of 4s epochs. After running the algorithms, the totality of the sleep measurements was checked for correctness and to further score epochs that could not be classified by the algorithm. For the visual scoring the different sleep phases were defined by the following criterion, as used by others [33–35]. Wakefulness is defined by an EMG activity which is high and variable and by medium EEG activity prominent in the beta (15–30 Hz) and gamma (30–100 Hz) ranges. During NREM sleep epochs low EMG activity is observed with a prominent delta (1–4 Hz) activity in the high EEG activity. During REM sleep very low EMG activity is characteristic accompanied by medium EEG activity with power concentrated in the theta (6–10 Hz) band [33]. Exemplary data for all three sleep stages can be seen in Fig. 1E for Wake, Fig. 1F for NREM and Fig. 1G for REM.

The visual check was done using an open-source toolbox for visual EEG analysis with some minor adaptations to customized the toolbox for our setup [36]. To facilitate the visual analysis, the collected data was band-pass filtered using a Butterworth filter with the cutoffs set at 0.5 Hz and 40 Hz respectively.

Based on sleep scoring, the macrostructure of the sleep was determined for a full day period, as well as for the light and the dark period separately. The bout number and the average bout duration of each sleep stage were also calculated. Lastly, the delta power during NREM and the theta power during REM were calculated as a marker of the microarchitecture of the sleep–wake behavior. This was done using the band power function of MATLAB. The cutoff values of 1 and 4.6 Hz for delta and 4 and 8 Hz for theta for determined using previous literature [37–39].

### RNA extraction

Brain hemispheres were divided into cerebellum, midbrain, and frontal sections. The brain tissue was placed in the appropriate volume of TRIzol® Reagent (1 mL per 100 mg tissue, Invitrogen) was homogenized (TissueRuptor II, Qiagen) for 20 s at full speed. RNA extraction was performed using an RNA Tissue Kit (PureLink RNA extraction kit, ThermoFischer; Universal RNA Tissue Kit). After samples were homogenized, one volume of 70% EtOH was added to the tissue homogenate. The mixture was thoroughly mixed by vortexing. Afterwards the mixture was transferred to the spin cartridge and centrifuged at 12,000×*g* for 15 s at room temperature. The flow-through was discarded and the spin cartridge was reinserted in the same collection tube. This step was performed until the whole sample was processed. In the next step 700 µL of Wash Buffer I was added and centrifuged at 12,000×*g* for 15 s. After the centrifugation, the flow-through as well as the collection tube were discarded, and the spin cartridge was placed on a new collection tube. Next, 500 µL of Wash Buffer II was added to the Spin Cartridge and centrifuged at 12,000×*g* for 15 s. The flow-though was discarded and the spin cartridge was reinserted in the same collection tube. This step was performed twice. After the washing steps, the spin cartridge was centrifuged at 12,000×*g* for 1 min at room temperature to dry the membrane. The spin cartridge was placed into a recovery tube and 40 µL of RNase-free water was added to the center of the spin cartridge and incubated for 1 min at room temperature to elute the RNA. After the incubation, the spin cartridge was centrifuged at 12,000×*g* for 2 min at room temperature. This step was done twice. The quality and quantity of the extracted RNA were assessed with NanoDrop™ One C (Thermo Scientific™) and stored at − 80 °C until further use.

### Primers

The primers which were used for the detection of LGTV strain TP21 were already described by [29] and were purchased from Microsynth (Balgach, Switzerland). LGTV forward primer 5′-TGTGTGGAGCGGCGATT-3′, LGTV reverse primer 5′-TAAGGGCGCGTTCCATCTC-3′ and LGTV probe 5′(FAM)-CTTGGCCCCCACACGAGTGGTG-(BHQ-1)-3′.

### One-step real-time PCR

A QuantStudio™ 6 Flex System with the QuantStudio™ Real-Time PCR software (Applied Biosystems) was used to conduct the one-step real-time PCR. RT-PCR was performed according to the manufactures instructions and as described in [29, 40]. The qRT-PCR conditions were as follows: 6.3 μL TaqMan Fast Virus 1-Step Master (ThermoFischer), 1 μL forward primer (10 μM), 1 μL reverse primer (10 μM), 0.6 μL of the probe stock (10 μM), 5 μL of sample containing the viral RNA. RNase-free water was used to adjust the volume to 25 μL.

The following parameters were used: reverse transcription at 50 °C for 10 min, polymerase activation at 95 °C for 5 min, 40 cycles of amplification with a two-step cycling of 3 s at 95 °C and 30 s at 60 °C. To determine the amount of virus particles in the brain parenchyma, a standard curve with a tenfold serial dilution of a virus vial with known concentration was used.

### Cytokine and chemokine in the CSF and serum

Five cytokines (IL-6, IFN-γ, MCP-1, IP-10, and RANTES) were assessed in the CSF and in the blood serum using a magnetic multiplex assay (MILLIPLEX MAP Rat Cytokine/Chemokine Magnetic Bead Panel–Immunology Multiplex Assay, Millipore) on a Bio-Plex 200 station (Bio-Rad Laboratories). For the three timepoints (day 4, day 9, and day 21 pi) 10 μL of CSF was diluted to a 50 ul final volume. For statistical purposes, values for the samples below the detection limit were calculated using the detection limit provided by the manufacturer multiplied by the dilution factor (IL-6 30.7 pg/mL, IFN-γ 6.2 pg/mL, MCP-1 9.0 pg/mL, IP-10 1.4 pg/mL, RANTES 1.3 pg/mL).

### Neurofilament in the CSF and the serum

Neurofilament light chain (NfL) in the CSF at different timepoints (day 4, 9, and 21 pi) and the serum (d4 and d9 pi) was analyzed using an automated ELISA-based microfluidic system (ELLA, ProteinSimple, San Jose, CA, USA) with the a 72 × 1 simpleplex NFL cartridge (Protein Simple) according to the manufacturer instructions, as previously described [41]. In brief, CSF samples were diluted 1:30 and serum samples 1:2 with the provided sample diluent and loaded on the cartridge wells. The cartridge architecture allows measurement of the samples in triplicate by splitting samples in different glass capillaries. Using the mean value of the signal intensities, samples concentrations corrected for dilution were calculated based on a lot-specific standard curve provided by the manufacturer.

### Statistical analysis

The distribution of the data was visually assessed and based on the Shapiro–Wilk test. Data was not normally distributed and therefore analyzed using the nonparametric Mann–Whitney U test. For the behavioral data, hypotheses were formulated based on previous results from animals and clinical data. One-tailed hypotheses were formulated before the collection of data and one-tailed tests were used. Statistical analyses for the sleep–wake behavior were conducted without pre-conceived hypotheses, using a two-tailed set-up. For the analysis of the three-hour bins, the comparisons were done using ANOVA. Values of p < 0.05 were considered as statistically significant. All of the statistical analyses were conducted using R-Studio, while the plots were done using Matlab 2019b.

## Results

### Confirmation of LGTV infection in brain tissue of infected animals

**Fig. 2:**
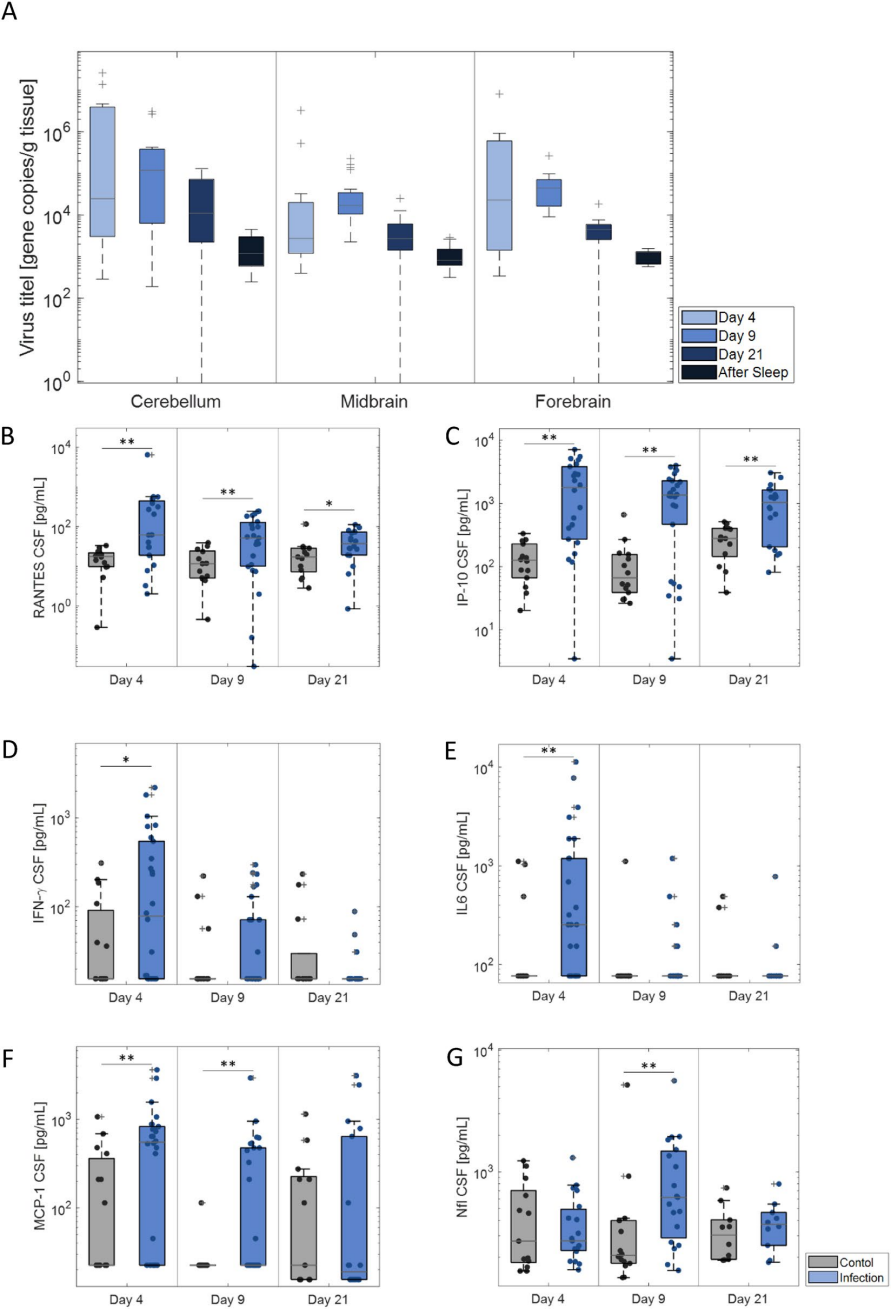
Viral load in brain tissue and increased CSF chemokines, cytokines and NFL in infected animals: **A** Virus titer at day 4 (n_cerebellum_ = 11; n_midbrain/forebrain_ = 12), day 9 (n_cerebellum_ = 13; n_midbrain/forebrain_ = 14), day 21 (n = 9) and after the sleep measurement (n = 6). **B**–**F** Chemokines and Cytokines assessed in the CSF of control and infected. **G** Nfl level assessed in CSF in control and infected animals, with significantly higher level in the infected animals at Day 9. †p < 0.1, *p < 0.05, **p < 0.01

The first step was to determine whether successful infection of LGTV took place in the brain tissue of the infected animals. The infection with the Langat virus was confirmed by one-step real-time PCR (Fig. 2A and Table 1). Four days after infection, animals showed the highest number of RNA copies in the cerebellum, followed by the forebrain and the midbrain. A similar pattern was observed at day 9, at day 21 post infection, and after sleep recording (day 42 post infection).

**Table 1:** Number of LGTV RNA copies**:** RNA copies measured at Day 4, Day 9, Day 21, and after the sleep measurement

	Cerebellum [copies/g tissue]	Midbrain [copies/g tissue]	Forebrain [copies/g tissue]
Day 4	4,300,000	270,000	960,000
Day 9	560,000	33,000	44,000
Day 21	34,000	4600	5200
After Sleep	1770	1140	1110

Overall, the number of RNA copies steadily declined over the investigated time points. For the control animals, no viral RNA was detected.

No difference in the weight or the weight gain could be observed between the control animals and the infected animals, as depicted in an additional figure (see Additional file 1). Survival was 100%, in both uninfected and infected animal groups.

### Markers of inflammation and neuronal damage are increased in the CSF of infected animals

In order to assess the level of inflammation and neuronal damage in infected animals, an array of markers was assessed, as these have been previously shown to increase in either TBEV or other models of encephalitis. Chemokines, cytokines and Neurofilament light chain (NfL), were assessed in the CSF of the animals at day 4, day 9, and day 21 pi (Fig. 2B–G and Table 2). The CSF levels of RANTES and IP-10 were significantly higher in infected animals compared to control animals at day 4, day 9, and day 21 pi (Fig. 2B and C). At day 4 pi IL-6 and IFN-γ were significantly higher in infected animals (Fig. 2D and E). Significant differences were also observed for MCP-1 at day 4 and day 9 pi (Fig. 2F).

**Table 2:** CSF concentrations of inflammatory parameters and NfL

Cytokine	Timepoint	Control [pg/mL]	Infection [pg/mL]	p-Value
RANTES	**Day 4**	**16.79 (n = 14)**	**503.18 (n = 20)**	**0.0033**
	**Day 9**	**14.54 (n = 13)**	**80.31 (n = 23)**	**0.0062**
	**Day 21**	**23.98 (n = 13)**	**45.39 (n = 18)**	**0.0216**
IL-6	**Day 4**	**236.94 (n = 15)**	**1363.22 (n = 26)**	**0.0082**
	Day 9	145.73 (n = 15)	142.99 (n = 28)	0.1816
	Day 21	131.69 (n = 13)	120.02 (n = 18)	0.6472
IFN-γ	**Day 4**	**68.13 (n = 15)**	**361.22 (n = 26)**	**0.0281**
	Day 9	39.68 (n = 15)	63.23 (n = 28)	0.1396
	Day 21	49.07 (n = 13)	22.25 (n = 18)	0.7477
MCP-1	**Day 4**	**224.61 (n = 15)**	**697.12 (n = 26)**	**0.0164**
	**Day 9**	**29.04 (n = 14)**	**306.09 (n = 28)**	**0.0076**
	Day 21	204.48 (n = 13)	460.53 (n = 18)	0.5255
IP-10	**Day 4**	**140.57 (n = 14)**	**2188.54 (n = 22)**	**0.0001**
	**Day 9**	**131.62 (n = 14)**	**1533.96 (n = 26)**	**0.0003**
	**Day 21**	**279.62 (n = 13)**	**1095.21 (n = 18)**	**0.0034**
NfL	Day 4	472.77 (n = 13)	401.47 (n = 19)	0.878
	**Day 9**	**633.50 (n = 14)**	**1065.21 (n = 19)**	**0.0083**
	Day 21	346.9 (n = 10)	392.7 (n = 10)	0.6232

Bold font represents significant table entries (p < 0.05)

CSF levels of NfL were similar between infected and controls animals at day 4 (401.47 ± 67.7 vs 472.77 ± 106.6 pg/mL) and 21 pi (392.7 ± 58.0 vs 346.6 ± 59.2 pg/mL). At day 9 (Fig. 2G), a significant (p = 0.0083) increase of NfL levels in the CSF was observed in infected animals (1065.2 ± 288.6 pg/mL) compared to the control animals (633.5 ± 353.1 pg/mL).

### Markers of inflammation and neuronal damage are not significantly increased in the serum of infected animals

As a next step it was assessed whether the changes of markers of inflammation observed in the CSF could also be detected in the blood. For this, the cytokines, chemokines, and NfL were also assessed in the serum. No significant difference between the two groups could be observed as it can be seen in an Additional file 2: table.

### Coordination and general locomotor activity are decreased in infected animals

**Fig. 3:**
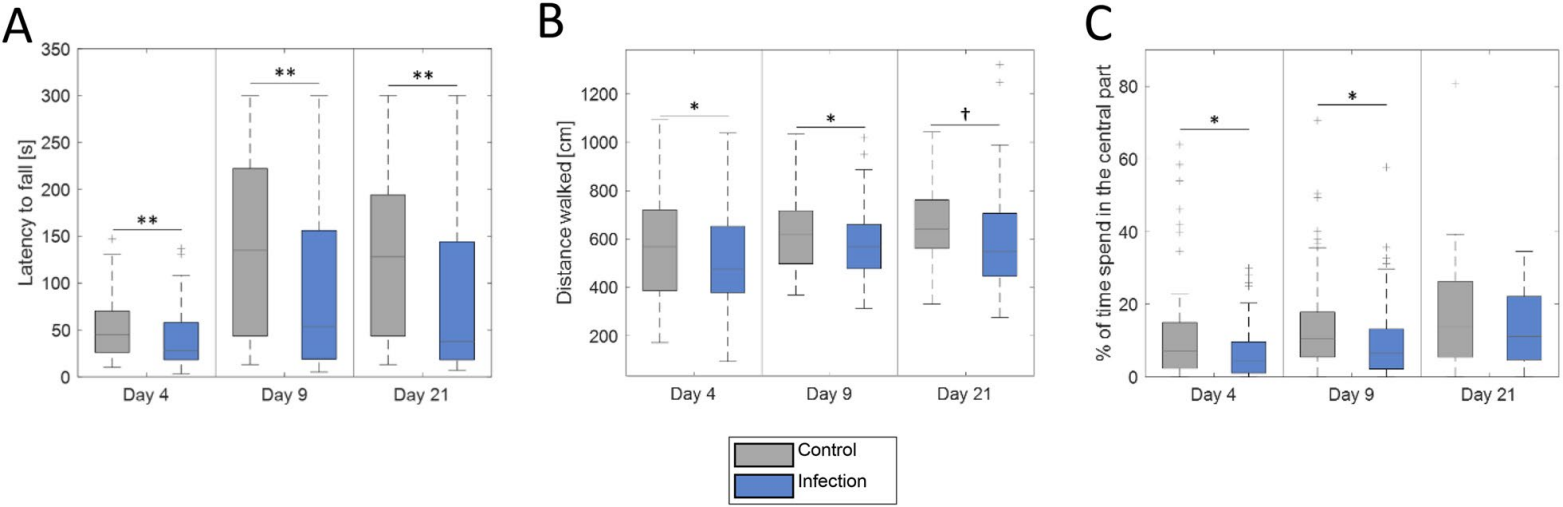
Reduced performance in the behavioral tests assessing motor coordination and locomotion following LGTV infection: **A** Rotarod test showing the latency to fall in seconds (day 4: n_control_ = 31, n_infection_ = 36; day 9: n_control_ = 22, n_infection_ = 26; day 21: n_control_ = 15, n_infection_ = 14), **B** and **C** Open field tests (day 4: n = 24; day 9: n = 20; day 21: n_control_ = 13, n_infection_ = 14) showing the average distance walked in cm and the percentage of the time spent in the center of the arena, respectively. †p < 0.1, *p < 0.05, **p < 0.01

It was then determined whether the deficits in motor coordination and function, which are commonly observed after TBEV infection in patients, can also be observed in the experimental model. In infected animals, a durable impairment in the Rotarod test was documented (Fig. 3A). A shorter latency to fall was determined in LGTV-infected animals compared to control animals at 4 days pi (39.31 ± 3.04 s vs. 51.47 ± 3.36 s, p = 0.0011), at 9 days pi (91.69 ± 15.50 s vs. 138.89 ± 15.77 s, p = 0.0055) and at 21 days pi (103.43 ± 16.26 s vs. 172.04 ± 17.11 s, p = 0.0037).

In the open field test, two parameters were determined. General locomotor activity was assessed by determining total walking distance. Infected animals moved less than the control animals. This resulted in a significant difference at day 4 (502.0 ± 2.9 cm vs. 579.7 ± 3.1 cm, p = 0.0196) and day 9 pi (584.9 ± 2.6 cm vs. 631.5 ± 2.6 cm, p = 0.0473) and a trend at day 21 pi (605.7 ± 5.7 cm vs. 656.9 ± 4.3 cm, p = 0.0646, Fig. 3B).

Furthermore, the exploratory behavior of the animals was assessed by determining the time spent in the center and the periphery of the arena (Fig. 3C). Control animals spent significantly more time in the center of the arena compared to infected animals at day 4 (6.28 ± 0.10% vs. 11.44 ± 0.19%, p = 0.0123) and day 9 pi (9.96 ± 0.18% vs. 14.44 ± 0.23%, p = 0.0101).

### An increased percentage of wake stage is observed after the LGTV infection

**Fig. 4:**
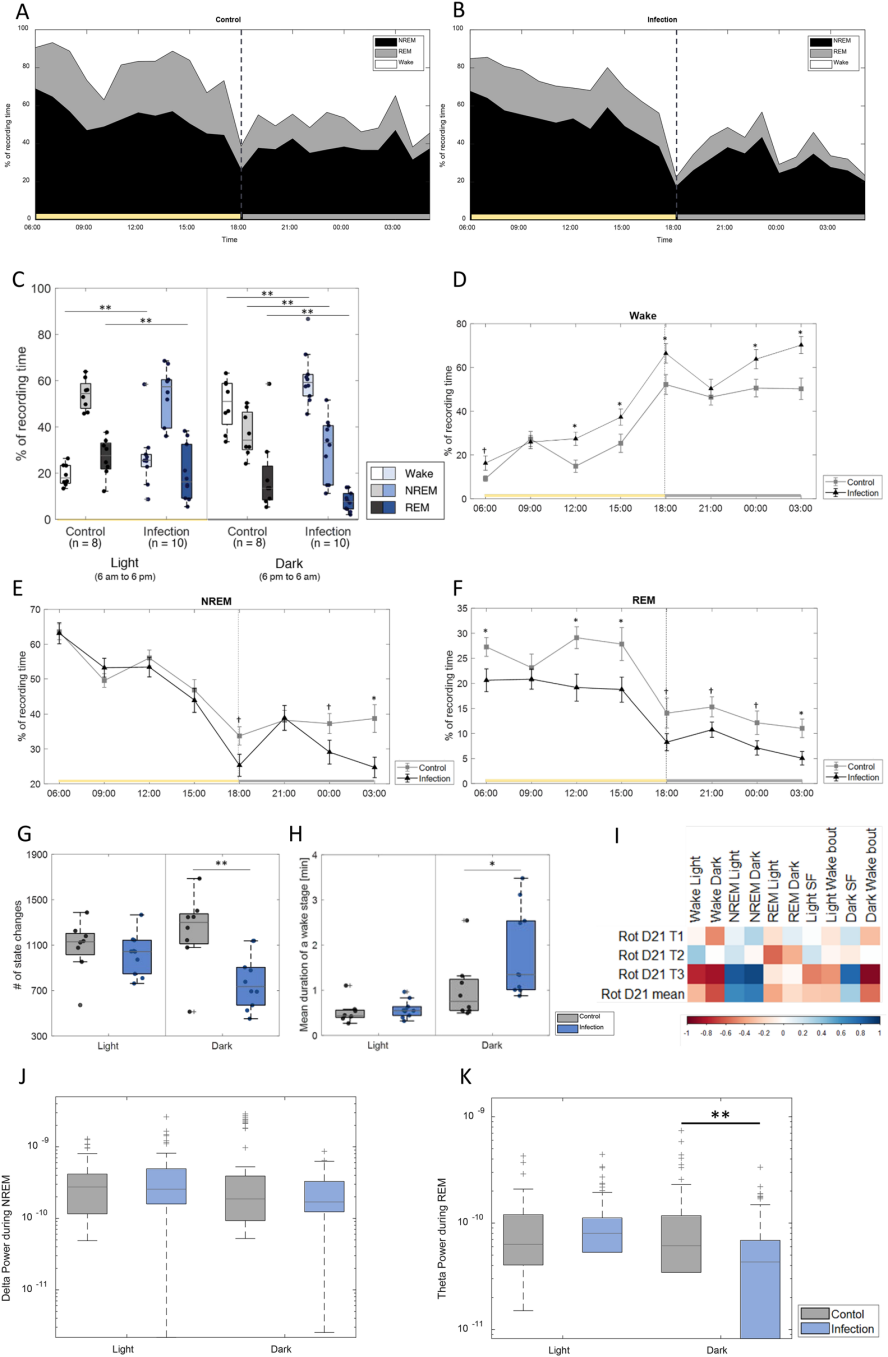
Extensive changes to the sleep structure determination by EEG/EMG after the LGTV infection: Sleep macrostructure of control (**A**) and infected (**B**) animals over 24 h. **C** Overview of the percentage in wake, NREM, and REM phases during light and dark period in control and infected animals. **D**–**F** Percentage of epochs displayed in 3 h bins for the wake, NREM, and REM phases, confirming that the infected animals spent more time awake than the control animals. **G** Mean number of state changes. **H** Mean duration of wake stage. **I** Correlation between sleep markers and the rotarod performance at Day 21. **J** Delta power during NREM in the light and dark period. **K** Theta power during REM for the light and dark period. n_control_ = 8, n_infection_ = 10; †p < 0.1, *p < 0.05, **p < 0.01

Since fatigue and sleep–wake disturbances are regularly mentioned as symptoms of the post-encephalitic syndrome, the study assessed the sleep–wake behavior as a long-term effect after experimental LGTV infection. Therefore, sleep macrostructure (percentage of time spent in wake, REM, and NREM phases) was assessed over a period of 24 h and sub-divided into the light and dark periods (Fig. 4A and 4B, respectively).

When assessing the total light period, infected animals spent significantly more time awake (26.74% for the infection and 19.17% for the control, p = 0.01) and less in REM sleep (19.83% vs. 26.83%, p = 0.0006) compared to the control animals, while NREM sleep was similar between the groups. During the dark period infected animals spent significantly more time awake (62.70% vs. 49.85%, p = 0.00002) and significantly less time in NREM (29.50% vs. 37.00%, p = 0.0047) as well as in REM sleep (7.80% vs. 13.14%, p = 0.0007, Fig. 4C).

Next, additional markers of sleep stages were assessed to thoroughly characterize the sleep–wake behavior. The differences in the different sleep stages were assessed in 3 h bins (Fig. 4D–F and Table 3 Sleep macrostructure for 3-h windows Table 3). A significant difference in the fragmentation of the sleep, as determined by the number of state changes, could be documented during the dark period, where the infected animals displayed a lower number of state changes than the control animals (776.5 ± 71.6 vs 1221.1 ± 112.4, p = 0.0085, Fig. 4G). This was not observed during the light phase.

**Table 3:** Sleep macrostructure for 3-h windows, n_control_ = 8, n_infection_ = 10

	Wake			NREM			REM		
	Control (%)	Infection (%)	p-value	Control (%)	Infection (%)	p-value	Control (%)	Infection (%)	p-value
06:00–09:00	9.19	16.27	0.08	63.56	63.14	0.92	**27.25**	**20.59**	**0.04**
09:00–12:00	27.32	25.94	0.77	49.56	53.24	0.31	23.11	20.82	0.50
12:00–15:00	**14.85**	**27.43**	**0.01**	56.05	53.43	0.50	**29.11**	**19.14**	**0.01**
15:00–18:00	**25.34**	**37.31**	**0.04**	46.83	43.94	0.55	**27.83**	**18.75**	**0.03**
18:00–21:00	**52.19**	**66.42**	**0.03**	33.75	25.31	0.06	14.07	8.27	0.09
21:00–24:00	46.44	50.34	0.51	38.25	38.90	0.89	15.31	10.76	0.07
00:00–03:00	**50.56**	**63.79**	**0.04**	37.29	29.09	0.09	12.15	7.12	0.07
03:00–06:00	**50.23**	**70.23**	**0.00**	**38.74**	**24.71**	**0.01**	**11.04**	**5.06**	**0.01**

Bold font represents significant table entries (p < 0.05)

The lower number of state changes in infected animals was accompanied by a longer duration of the wake bouts in the infected animals during the dark phase as compared to control animals (1.83 ± 0.29 min vs 1.02 ± 0.23 min, p = 0.0434, Fig. 4H), while no significant difference in NREM and REM bout durations could be detected as depicted in an additional figure (see Additional file 3).

### A higher percentage of wake correlated with lower performance in behavioral tests in infected animals

Next, it was evaluated whether a correlation between the results of the behavioral testing and the sleep analysis exists. A strong correlation could be observed between the last trial of the Rotarod at Day 21 and sleep parameters in the group of infected animals (Fig. 4I). As such significant correlations between the Rotarod performance and Wake, NREM in both lightning conditions were observed. Wake was negatively correlated with the latency to fall (r = − 0.77, p = 0.009 for Light and r = − 0.83, p = 0.003 for Dark), while NREM was positively correlated (r = 0.84, p = 0.002 for Light and r = 0.90, p = 0.0003). Furthermore, a positive correlation was found between the last trial of the Rotarod at Day 21 and the sleep fragmentation during dark (r = 0.78, p = 0.007) as well as a negative correlation with the wake bout length during dark (r = − 0.92, p = 0.0002).

### LGTV infections decreases theta spectral power during REM sleep phases, while not affecting delta power during NREM sleep

As a last step, we assessed whether differences in sleep microstructure between the control animals and the infected animals were present. No significant difference could be found for the delta power during NREM between the groups (Fig. 4J). For theta power during the REM sleep, a significant difference was found between the groups during the dark period (p = 0.00036, Fig. 4K) with a higher delta power measured for the control animals. The same pattern was not seen during the light period (p = 0.354, Fig. 4K).

## Discussion

In the present study, we investigated how LGTV-induced encephalitis influences sleep behavior in juvenile rats. Changes to sleep–wake behavior after TBE infections have been previously described in patients, yet very rarely assessed using objective measurement techniques. The current literature has identified changes to sleep–wake behavior after TBE in up to 73.9% of children suffering from fatigue while 27.4% of adults have reported extensive daytime sleepiness or somnolence. A recent in-depth literature review indeed confirm that sleep wake and circadian disorders are a relevant issue in TBE-affected patients [42].

To the best of our knowledge, this is the first study assessing the consequences of neuroinfection caused by LGTV in wildtype rats over such an extensive time, and with such a large sample, while combining inflammatory parameters, behavior results with regards to locomotion and motor coordination and the assessment of sleep–wake behavior.

The major novelty of this study is the identification and characterization of sleep–wake disorders after LGTV infection. Sleep–wake behavior was assessed for 24 consecutive hours, encompassing a full light and dark cycle. We observed that the control animals spent significant less time awake and significantly more time in REM sleep in the light period. In the dark period, the control animal spent significantly less time awake and therefore significantly more time in NREM and REM sleep than the infected animals.

Infected animals had a reduced number of state changes during the dark period compared to the control animals. Additionally, the duration of a wake bout in the dark phase was elongated in the infected animals.

After TBE, the most consistently reported parameter related to sleep–wake disturbance is fatigue, with a significantly higher number of patients complaining from fatigue compared to control groups [17–19, 43]. Fatigue is assessed throughout the day and therefore represents a sleep–wake disturbance seen during the active phase of the patients [44].

The results in the experimental model are in line with these observations of sleep–wake disturbances in patients during the active phase. Similar to fatigue in patients, sleep–wake disorders in rats are also found during the active phase, namely, during the dark period. It has been debated in patients, whether fatigue is a direct physiological response to TBEV infection or whether this is a more subjective complaint, potentially arising from indirect changes to the daily routine due to the TBEV infection.

Further, the experimental model revealed clear changes in the sleep macrostructure which were measured with objective parameters compared to a matched control group, under identical conditions. Therefore, our study gives a first indication, that the sleep–wake disorders could be a result of the infection itself and not caused by a change of the patient’s circumstances. In patients, there is limited data that support a direct effect of TBEV infection on the sleep–wake disorders. MRI studies from patients show a clustering of virus-induced lesions in the thalamus [45, 46]. Due to the importance of the thalamus in the induction, stabilization, and termination of sleep [47], the hypothesis, that this affected structure is responsible for sleep–wake disturbance consecutive to TBE, is not unlikely. A prolongation of the wake bouts compared to the controls during the dark phase in the present experimental model further support this hypothesis. Lesion studies targeting the thalamus in experimental models have not yielded substantial changes in the sleep–wake behavior [48]. In thalamic stroke patients, however, a change in the sleep–wake behavior has been reported, including modifications in arousal and in the production of NREM sleep [47, 49, 50].

Extensive changes in sleep macrostructure, as observed in infected animals, could not be documented in a recent case–control study assessing similar parameters in TBE patients [18]. This discrepancy could be caused by differences in the experimental setup, since the clinical study only conducted measurement during a single night, while data were collected for 24 h in the experimental study. Further, in animals, the study was conducted at a controlled, constant time, (i.e., within weeks after the infection), while in patients, sleep was assessed on average 2 years after the infection. Furthermore, even though the cases and controls were similar regarding age, sex, and other factors in the clinical study, the experimental study compared sibling animals, potentially constituting a more appropriate control group. It should additionally be mentioned that, even when the polysomnography (PSG) records did not detect significant differences, TBE patients reported statistically more sleep-related functional impairment on the Functional Outcome of Sleep Questionnaire (FOSQ) compared with the controls.

Changes to the sleep microarchitecture were also observed, with a significant decrease in theta power during REM observed during the dark period in the infected animals. Theta power has been hypothesized to be involved in emotional memory consolidation and processing [51]. Furthermore, it has been seen in other animal models, that theta power is reduced in a tau model of neurodegeneration [52] or in animals that were subjected to chronic social defeat stress [53]. To the best of our knowledge, sleep microarchitecture has never been assessed after tick-borne encephalitis, either experimentally or in patients. 

LGTV was detected in the brain parenchyma at each assessed timepoint. At day 4 pi the highest viral load was detected. The viral load decreased over time with the lowest viral load measured after the sleep recording. A similar trend of decrease of viral load over time was seen in previous literature using experimental models [29, 54].

Five cytokines were assessed in the CSF and in the serum. In infected animals, IFN-γ and IL-6 levels were significantly higher at day 4 pi than in the control animals. The MCP-1 levels in the CSF were significantly higher in infected animals at day 4 and day 9 pi. The level of IP-10 and RANTES in CSF were significantly higher in infected animals in all three assessed timepoints.

The chemokine and cytokine responses are similar to those that can be observed in patients or other experimental models. RANTES/CCL5 was significantly increased in the CSF of TBE patients at the hospital admission [55, 56] as well during as up to 16 days after the admission [55, 57]. Induction of RANTES expression was also observed in the brain tissue of infected mice after TBEV infection [27].

In the acute phase of TBE CSF IL-6 levels were significantly increased in adult TBE patients compared to a control group [58], and was proposed as a useful prognostic tool [59, 60]. After 10 to 12 days the IL-6 levels return to levels comparable to those of control patients [58].

Increased CSF levels of IFN-γ were also seen in TBE patients with the highest level shortly after the beginning of the infection [61, 62]. Furthermore, children with significantly increased IFN-γ and IL-6 levels in the acute phase in the CSF developed sequela in the later phase [63].

TBE patients displayed a significant increased level of MCP-1/CCL2 in the CSF at hospital admission [55, 64] and a significant increase of MCP-1/CCL2 was also shown in the brain of TBEV infected animals in [65, 66].

A significantly increased level of IP-10/CXCL10 in CSF adults and children patients with TBE was described compared to the control group [55] at the admission to the hospital as well as 3 weeks later [67]. The increase of IP-10/CXCL10 over time was shown also in an experimental model, showing significantly increased levels in the brain starting from day 5 post infection [65].

Previous literature has indicated that in children, the extend of the inflammatory response can be predictive of the behavioral impairments. For example, the level of IL-6 during the acute phase of TBE has been proposed as a predictor for the development of sequelae in children [63]. Data of the present study did however not reveal that a cytokine or chemokine can reliably predict behavior with regards to motor coordination as shown in two additional tables (see Additional file 4).

Sleep disturbances have been previously associated with enhanced cytokine levels of e.g., IL-6 and IP10 in patients [68, 69]. However, in contrast to these studies in patients, sleep–wake disorders were determined 21 days after infection in the animals of the present study, at a time when most of the cytokines returned to basal levels. It is therefore unlikely that inflammatory is a direct driver of sleep disturbance. Furthermore, no significant contribution of the IP-10 cytokine levels measured at the earlier time points on the change in sleep–wake behavior could be observed [see Additional file 5, additional Fig. 3]. This was assessed using a one-way ANOVA.

We found a significant increased level of neurofilament light chain (NfL) in the CSF of infected animals at day 9 pi. NfL is a cytoskeletal protein that is found specifically in myelinated subcortical axons. Increased CSF level of NfL is indicative of axonal damage and neuronal death and has been reported in a wide variety of CNS disorders, such as multiple sclerosis, Parkinson’s disease, and amyotrophic lateral sclerosis [70]. Furthermore, an increase of NfL level in the CSF, as well as the serum, has been observed after CNS infections with varicella-zoster virus [71]. It has further been seen in pneumococcal meningitis in an experimental model [72] as well as for childhood bacterial meningitis [73]. To date, CSF levels of NfL after TBE have been described in one study. The CSF was sampled at a mean duration from neurological symptoms of 4.2 days. TBE patients showed a higher NfL level in the CSF compared to the control indicating a higher level of neuronal injury [74].

When assessing the behavior of the animals, we found, that infected animals showed a significant reduction of balance and motor coordination at all the measurement times.

Human histopathological studies show an affinity of the TBE virus for the basal ganglia, mesencephalon, diencephalon, medulla oblongata, pons, Purkinje cells in the cerebellum as well as the anterior horn cells in the spinal cord [4, 11, 75, 76]. In the present study, a high concentration of LGTV was documented in the cerebellum. Since the cerebellum is involved in motor coordination and balance [77], viral infection in this brain area could explain the reduction of locomotion and coordination seen in infected animals. This would be in line with ataxia, often documented in clinical analysis of TBE patients, with an occurrence ranging from 6 to 72% [13, 14, 16, 45, 78].

In the open field, we saw significant changes in general locomotor activity and the willingness to explore a new environment after the infection. This effect was seen at day 4 and day 9 after the infection. These results are in line with previous results using the same animal model [29]. The open field test is also used to determine the anxiety-like behavior in animals [79]. Infected animals showed anxiety-like behavior with less exploration of the center of the arena. Increased anxiety and depression have been reported in patients in several previous studies [17, 80, 81]. This behavior has not been reported in an animal model of TBE infection, and a previous study even reported reduced anxiety-like behavior in a similar model in wildtype mice after infection [82].

A limited number of clinical studies have assessed the relationship between infectious diseases and sleep–wake disorders, and it stays unclear whether changes in sleep–wake behavior could be caused by the virus itself or rather by the immune answer to the infection. Several viral infections, such as Polio, HIV, Hepatitis C, Varicella zoster, and Zika have been associated with the development of sleep–wake disorders. This is best documented for HIV-infected patients, that develop sleep–wake disorders already during the asymptomatic phase of the disease. During HIV, these perturbances are most likely due to a dysregulated immune response rather than caused by the patient's medication [83, 84]. Using actigraphy, a significant increase in sleep onset latency (SOL) has been observed in people living with HIV compared to a gender- and age-matched control group [85]. Additionally, in a polysomnographic study of people 60 days after a SARS-CoV-2 infection, a mean SOL of approximately 90 min was observed [86], which is compared to 20 min in age-matched controls [87]. The increase in SOL could be compared to the longer wake bout period which has been observed in our study in the infection group.

A limitation of this study is, that the current EEG/EMG setup did not have a sufficiently high spatial resolution to capture the local aspects of the microarchitecture of sleep e.g., in different brain regions. As such it is difficult to judge how and if the local phenomena of sleep in the infected animals are still maintained or whether these are also disrupted.

As with other studies on long-term outcomes, Langat Virus was selected as a model for TBEV, as TBEV is associated with a high lethality in laboratory rodents [88, 89] that precludes the assessment of long-term effects.

## Conclusion

This study extends previous research using the present experimental model and further characterizes features consistent with a post encephalitis syndrome observed in patients after TBE. A reduction of motor coordination and spontaneous locomotion could be documented up to 21 days after infection. Moreover, anxiety-like behavior was observed in the infected animals, a symptom often described in TBE patients.

We also thoroughly characterized sleep–wake behavior after the infection, with significant differences between the infected animals and control animals, mainly during the time rats are active (dark period). This is comparable to the often-reported daytime sleep–wake disorders in TBEV patients, such as fatigue.

However, more research in patients as well as in animals is needed to better understand the underlying causes of sleep–wake disorders after TBE. Moreover, the effect of sleep–wake disorders on the outcome after the TBEV infection should be assessed to evaluate whether treating patients for sleep–wake disorders would be a helpful tool during their neurorehabilitation.

## Supplementary Information

**Additional file 1. **The weight data from the control and the infection group are depicted. Additional Fig. S1. Weight data**:** Mean weight data and standard deviation over time for the control group and infection group, Day 0 to 4: n_infection_ = 54, n_control_ = 43; Day 5 to 9: n_infection_ = 42, n_control_ = 36; Day 10 to 21: n_infection_ = 28, n_control_ = 26.

**Additional file 2. **Displays the results from the chemokines, cytokines, and NfL as measured in the blood serum. Additional Table S1: Blood serum concentrations of inflammatory parameters and NfL.

**Additional file 3. **Depicts the results with respect to the bout length during NREM and REM. Additional Fig. S2: Bout length during NREM and REM: A) NREM bout length for the infection and the control group during the Light and Dark period. No significant difference was found during the Light period (p = 0.97) and Dark period (p = 0.27). B) REM bout length for the infection and control group during the Light and Dark period. No difference was found during either period (p = 0.97 for both periods), n_infection_ = 10, n_control_ = 8.

**Additional file 4. **The results of the analysis of the correlation between the chemokines and cytokines and the motor coordination. This file contains two tables to qualify the correlation: Additional Table S2: p-Value of the correlations between Chemokines and cytokines and Rotarod behavior in the infection group. And Additional Table S3. rho-Value of the correlations between Chemokines and cytokines and Rotarod behavior in the infection group.

**Additional file 5. **Shows the analysis into the potential of using IP10 levels as predictor for sleep–wake behavior. Additional Fig. S3. Results on the analysis of IP-10 level as a contributor for the sleep–wake behavior: A) Results for Wake during Light B) Wake during Dark, C) NREM during the Light Period, D) NREM during Dark, E) REM during Light and F) REM during Dark. None of the analysis revealed any influence of the cytokine level (IP) or the cytokine level at a specific day (IP:Day).

## Data Availability

The datasets used and/or analyzed during the current study are available from the corresponding author on reasonable request.

## Abbreviations

BHQ-1: Black Hole Quencher-1
CNS: Central Nervous System
CSF: Cerebrospinal Fluid
EEG: Electroencephalogram
ELISA: Enzyme-linked immunosorbent assay
EMG: Electromyogram
FAM: Fluorescein Phosphoramidite
FOSQ: Functional Outcome of Sleep Questionnaire
HIV: Human Immunodeficiency Virus
IFN-γ: Interferon-gamma
IL-6: Interleukin-6
IP-10/CXCL10: Interferon-gamma induced protein-10/C-X-C motif chemokine Ligand 10
LGTV: Langat Virus
MCP-1/CCL2: Monocyte Chemoattractant Protein-1/ C–C Motif Chemokine Ligand 2
NfL: Neurofilament light chain
NREM: Non-Rapid Eye Movement
PBS: Phosphate Buffered Saline
PC12 cells: Pheochromocytoma 12 cells
PCR: Polymerase Chain Reaction
pi: Post infection
PSG: Polysomnography
RANTES/CCL5: Regulated on Activation, Normal T Expressed and Secreted/ C–C motif chemokine ligand 5
REM: Rapid Eye Movement
s.c.: Subcutaneous
SARS-CoV-2: Severe Acute Respiratory Syndrome-Coronavirus type-2
SOL: Sleep onset latency
TBE: Tick-borne encephalitis
TBEV: Tick-borne encephalitis virus
TBEV-Eur: Tick-borne encephalitis virus European Subtype
TBEV-FE: Tick-borne encephalitis virus Far Eastern Subtype
TBEV-Sib: Tick-borne encephalitis virus Siberian Subtype
