# Supplementary material for: Tick-borne encephalitis affects sleep–wake behavior and locomotion in infant rats
Source: Cell Biosci. 2022 Aug 2;12:121. doi: 10.1186/s13578-022-00859-7 (PMC9344439; doi:10.1186/s13578-022-00859-7)

Additional Figure 1 Weight data: Mean weight data and standard deviation over time for the control group and infection group, Day 0 to 4: $n_{infection} = 54$, $n_{control} = 43$; Day 5 to 9: $n_{infection} = 42$, $n_{control} = 36$; Day 10 to 21: $n_{infection} = 28$, $n_{control} = 26$

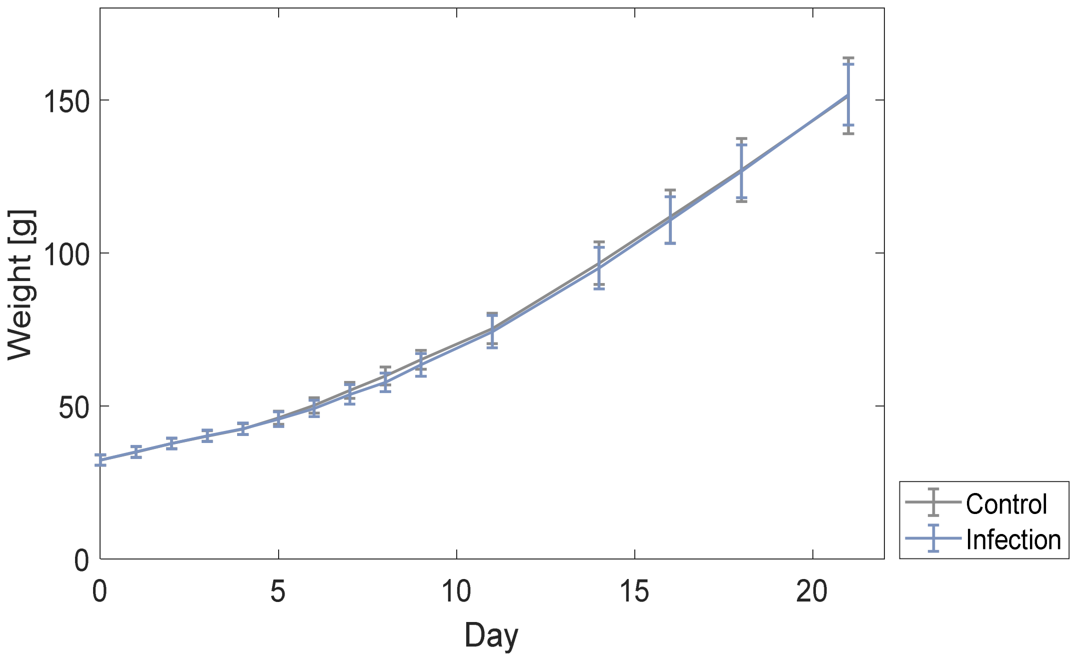

Supplement: Supplementary file 1 — Additional file 1. The weight data from the control and the infection group are depicted. Additional Fig. S1. Weight data: Mean weight data and standard deviation over time for the control group and infection group, Day 0 to 4: ninfection = 54, ncontrol = 43; Day 5 to 9: ninfection = 42, ncontrol = 36; Day 10 to 21: ninfection = 28, ncontrol = 26. [file 13578_2022_859_MOESM1_ESM.pdf]
