# Supplementary material for: Tick-borne encephalitis affects sleep–wake behavior and locomotion in infant rats
Source: Cell Biosci. 2022 Aug 2;12:121. doi: 10.1186/s13578-022-00859-7 (PMC9344439; doi:10.1186/s13578-022-00859-7)
Supplement: Supplementary file 2 — Additional file 2. Displays the results from the chemokines, cytokines, and NfL as measured in the blood serum. Additional Table S1: Blood serum concentrations of inflammatory parameters and NfL. [file 13578_2022_859_MOESM2_ESM.pdf]

Additional Table 1: Blood serum concentrations of inflammatory parameters and NfL.

Cytokine	Timepoint	Control [pg/mL]	Infection [pg/mL]	p-Value
**RANTES**	Day 4	281.085 (n = 6)	1031.405 (n = 6)	0.24
	Day 9	813.435 (n = 6)	202.6 (n = 8)	0.23
	Day 21	11.45 (n = 6)	4.21 (n = 8)	0.66
**IL-6**	Day 4	510.365 (n = 6)	355.355 (n = 6)	1.00
	Day 9	61.4 (n = 6)	61.4 (n = 8)	0.62
	Day 21	61.4 (n = 6)	61.4 (n = 8)	0.31
**IFN-γ**	Day 4	347.72 (n = 6)	355.625 (n = 6)	0.74
	Day 9	374.33 (n = 6)	44.59 (n = 8)	0.10
	Day 21	12.4 (n = 6)	12.4 (n = 8)	0.98
**MCP-1**	Day 4	359.905 (n = 6)	2693.03 (n = 6)	0.19
	Day 9	2127.475 (n = 6)	1319.16 (n = 8)	0.98
	Day 21	624.305 (n = 6)	382.01 (n = 8)	0.73
**IP-10**	Day 4	254.85 (n = 6)	725.005 (n = 6)	0.06
	Day 9	134.89 (n = 6)	68.62 (n = 8)	0.41
	Day 21	39.15 (n = 5)	7.21 (n = 8)	0.95
**NfL**	Day 4	77.1 (n = 3)	87.9 (n = 4)	0.85
	Day 9	58.4 (n = 3)	54.05 (n = 4)	1
