# Supplementary material for: Tick-borne encephalitis affects sleep–wake behavior and locomotion in infant rats
Source: Cell Biosci. 2022 Aug 2;12:121. doi: 10.1186/s13578-022-00859-7 (PMC9344439; doi:10.1186/s13578-022-00859-7)

**Additional Figure 2: Bout length during NREM and REM**: A) Shows the NREM bout length for the infection and the control group during the Light and Dark period. No significant difference was found during the Light period (p = 0.97) and Dark period (p = 0.27). B) REM bout length for the infection and control group during the Light and Dark period. No difference was found during either period (p = 0.97 for both periods).

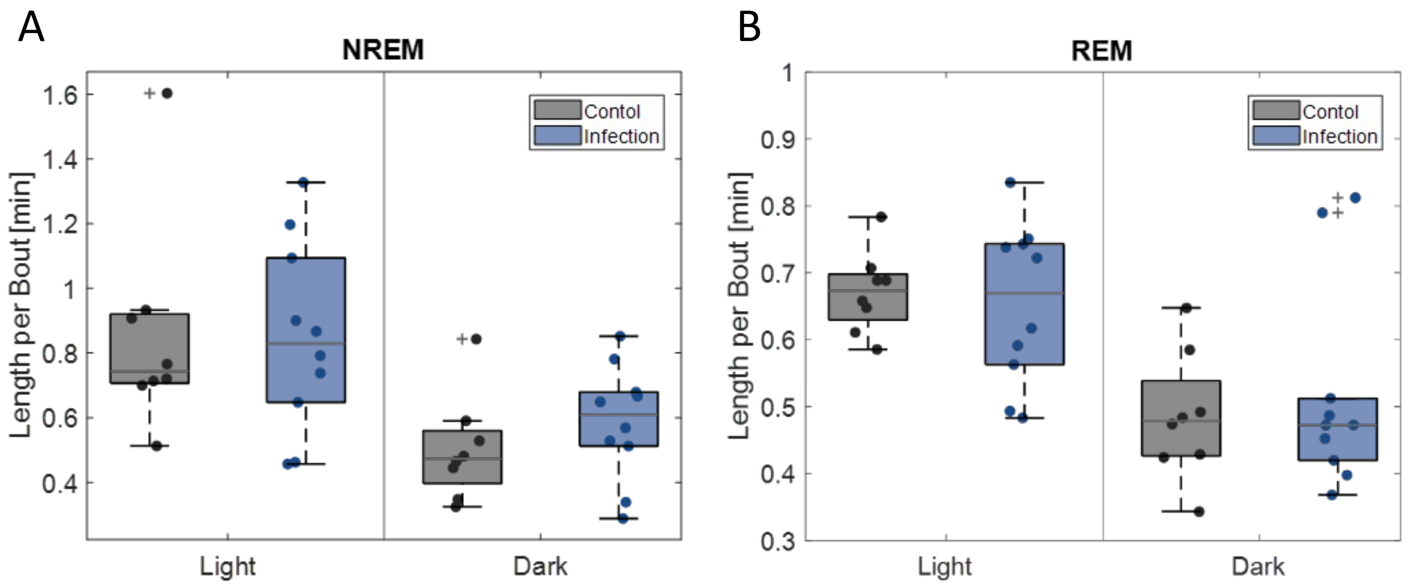

Supplement: Supplementary file 3 — Additional file 3. Depicts the results with respect to the bout length during NREM and REM. Additional Fig. S2: Bout length during NREM and REM: A) NREM bout length for the infection and the control group during the Light and Dark period. No significant difference was found during the Light period (p = 0.97) and Dark period (p = 0.27). B) REM bout length for the infection and control group during the Light and Dark period. No difference was found during either period (p = 0.97 for both periods), ninfection = 10, ncontrol = 8. [file 13578_2022_859_MOESM3_ESM.pdf]
