# Supplementary material for: Tick-borne encephalitis affects sleep–wake behavior and locomotion in infant rats
Source: Cell Biosci. 2022 Aug 2;12:121. doi: 10.1186/s13578-022-00859-7 (PMC9344439; doi:10.1186/s13578-022-00859-7)
Supplement: Supplementary file 4 — Additional file 4. The results of the analysis of the correlation between the chemokines and cytokines and the motor coordination. This file contains two tables to qualify the correlation: Additional Table S2: p-Value of the correlations between Chemokines and cytokines and Rotarod behavior in the infection group. And Additional Table S3. rho-Value of the correlations between Chemokines and cytokines and Rotarod behavior in the infection group. [file 13578_2022_859_MOESM4_ESM.pdf]

Additional Table 2: p-Value of the correlations between Chemokines and cytokines and Rotarod behavior in the infection group.

p-Value

	Day 4 (n = 13)					Day 9 (n = 22)					Day 21 (n = 11)				
	RANTES	IP10	MCP-1	IFN-γ	IL-6	RANTES	IP10	MCP-1	IFN-γ	IL-6	RANTES	IP10	MCP-1	IFN-γ	IL-6
Rot D4 T1	0.64	1.00	0.19	0.23	0.23	0.07	0.02	0.62	0.53	0.61	0.41	0.77	0.35	0.16	0.43
Rot D4 T2	0.27	0.71	0.96	0.69	0.09	0.18	0.94	0.21	0.37	0.68	0.41	0.09	0.51	0.32	0.75
Rot D4 T3	0.96	0.03	0.48	0.58	0.69	0.98	0.72	0.50	0.39	0.38	0.62	0.92	0.64	0.13	0.67
Rot D4 mean	0.58	0.26	0.31	0.78	0.31	0.31	0.56	0.96	0.84	0.52	0.78	0.52	0.77	0.12	0.74
Rot D9 T1	0.52	0.99	0.44	0.02	0.00	0.10	0.41	0.43	0.82	0.82	0.69	0.65	0.43	0.53	1.00
Rot D9 T2	0.84	0.95	0.67	0.50	0.98	0.09	0.33	0.82	0.18	0.46	0.32	0.50	0.46	0.59	0.77
Rot D9 T3	0.06	0.56	0.95	0.06	0.63	0.73	0.63	0.54	0.31	0.53	0.61	0.50	0.02	0.04	0.56
Rot D9 mean	0.82	0.88	0.87	0.06	0.04	0.27	0.41	0.79	0.18	0.47	0.50	0.42	0.45	0.64	1.00
Rot D21 T1	0.64	1.00	0.19	0.23	0.23	0.07	0.02	0.62	0.53	0.61	0.41	0.77	0.35	0.16	0.43
Rot D21 T2	0.27	0.71	0.96	0.69	0.09	0.18	0.94	0.21	0.37	0.68	0.41	0.09	0.51	0.32	0.75
Rot D21 T3	0.96	0.03	0.48	0.58	0.69	0.98	0.72	0.50	0.39	0.38	0.62	0.92	0.64	0.13	0.67
Rot D21 mean	0.58	0.26	0.31	0.78	0.31	0.31	0.56	0.96	0.84	0.52	0.78	0.52	0.77	0.12	0.74

Additional Table 3: rho-Value of the correlations between Chemokines and cytokines and Rotarod behavior in the infection group.

rho

	Day 4 (n = 13)					Day 9 (n = 22)					Day 21 (n = 11)				
	RANTES	IP10	MCP-1	IFN-γ	IL-6	RANTES	IP10	MCP-1	IFN-γ	IL-6	RANTES	IP10	MCP-1	IFN-γ	IL-6
Rot D4 T1	-0.16	0.00	0.43	-0.39	-0.39	0.44	0.54	-0.13	0.16	0.13	0.21	0.08	-0.24	0.35	0.21
Rot D4 T2	-0.36	-0.13	0.02	-0.13	-0.54	0.33	0.02	0.31	0.23	0.11	-0.22	-0.42	-0.17	-0.25	0.08
Rot D4 T3	-0.02	-0.65	0.24	0.19	-0.13	-0.01	-0.09	-0.17	-0.22	0.22	0.13	0.03	0.12	0.38	0.11
Rot D4 mean	-0.19	-0.37	0.33	-0.09	-0.34	0.25	0.15	-0.01	0.05	0.16	0.07	-0.17	-0.08	0.40	0.09
Rot D9 T1	0.17	0.00	0.21	0.57	0.68	0.37	0.19	0.18	-0.05	0.05	0.14	0.15	-0.26	-0.21	0.00
Rot D9 T2	0.05	-0.02	0.12	-0.18	0.01	0.38	0.22	0.05	-0.30	-0.17	0.33	-0.23	0.25	0.18	0.10
Rot D9 T3	-0.48	-0.16	-0.02	0.49	0.13	0.08	0.11	0.14	-0.23	-0.15	0.17	-0.23	0.67	0.63	0.20
Rot D9 mean	-0.06	-0.04	0.04	0.47	0.52	0.25	0.19	0.06	-0.30	-0.17	0.23	-0.27	0.25	0.16	0.00
Rot D21 T1	-0.16	0.00	0.43	-0.39	-0.39	0.44	0.54	-0.13	0.16	0.13	0.21	0.08	-0.24	0.35	0.21
Rot D21 T2	-0.36	-0.13	0.02	-0.13	-0.54	0.33	0.02	0.31	0.23	0.11	-0.22	-0.42	-0.17	-0.25	0.08
Rot D21 T3	-0.02	-0.65	0.24	0.19	-0.13	-0.01	-0.09	-0.17	-0.22	0.22	0.13	0.03	0.12	0.38	0.11
Rot D21 mean	-0.19	-0.37	0.33	-0.09	-0.34	0.25	0.15	-0.01	0.05	0.16	0.07	-0.17	-0.08	0.40	0.09
