# Supplementary material for: Tick-borne encephalitis affects sleep–wake behavior and locomotion in infant rats
Source: Cell Biosci. 2022 Aug 2;12:121. doi: 10.1186/s13578-022-00859-7 (PMC9344439; doi:10.1186/s13578-022-00859-7)
Supplement: Supplementary file 5 — Additional file 5. Shows the analysis into the potential of using IP10 levels as predictor for sleep–wake behavior. Additional Fig. S3. Results on the analysis of IP-10 level as a contributor for the sleep–wake behavior: A) Results for Wake during Light B) Wake during Dark, C) NREM during the Light Period, D) NREM during Dark, E) REM during Light and F) REM during Dark. None of the analysis revealed any influence of the cytokine level (IP) or the cytokine level at a specific day (IP:Day). [file 13578_2022_859_MOESM5_ESM.pdf]

Additional Figure 3: Results on the analysis of IP-10 level as a contributor for the sleep-wake behaviour: A) Results for Wake during Light B) Wake during Dark, C) NREM during the Light Period, D) NREM during Dark, E) REM during Light and F) REM during Dark
None of the analysis revealed any influence of the cytokine level (IP) or the cytokine level at a specific day (IP:Day)

A

```
> fm1 <- aov(Wake_Light ~ IP * Day, data = Wake)
> summary(fm1)
            Df Sum Sq Mean Sq F value Pr(>F)
IP           1  183.9  183.86   1.051  0.332
Day          1  195.6  195.62   1.118  0.318
IP:Day       1   57.8   57.81   0.331  0.579
Residuals    9 1574.1  174.90
```

B

```
> fm2 <- aov(Wake_Dark ~ IP * Day, data = Wake)
> summary(fm2)
            Df Sum Sq Mean Sq F value Pr(>F)
IP           1  227.3  227.25   1.173  0.307
Day          1    9.5    9.50   0.049  0.830
IP:Day       1   60.5   60.53   0.312  0.590
Residuals    9 1744.1  193.79
```

C

```
> fm3 <- aov(NREM_Light ~ IP * Day, data = NREM)
> summary(fm3)
            Df Sum Sq Mean Sq F value Pr(>F)
IP           1   89.6   89.58   0.449  0.520
Day          1   10.8   10.82   0.054  0.821
IP:Day       1    0.3    0.35   0.002  0.968
Residuals    9 1795.6  199.51
```

D

```
> fm4 <- aov(NREM_Dark ~ IP * Day, data = NREM)
> summary(fm4)
            Df Sum Sq Mean Sq F value Pr(>F)
IP           1  185.2  185.22   1.148  0.312
Day          1    1.2    1.19   0.007  0.933
IP:Day       1   12.0   12.04   0.075  0.791
Residuals    9 1451.4  161.27
```

E

```
> fm5 <- aov(REM_Light ~ IP * Day, data = REM)
> summary(fm5)
            Df Sum Sq Mean Sq F value Pr(>F)
IP           1   16.8   16.77   0.130  0.727
Day          1  114.4  114.43   0.886  0.371
IP:Day       1   67.1   67.10   0.520  0.489
Residuals    9 1162.4  129.15
```

F

```
> fm6 <- aov(REM_Dark ~ IP * Day, data = REM)
> summary(fm6)
            Df Sum Sq Mean Sq F value Pr(>F)
IP           1   2.15   2.147   0.132  0.725
Day          1   3.96   3.959   0.243  0.634
IP:Day       1  18.58  18.584   1.142  0.313
Residuals    9 146.51  16.279
```
